# Correlates of alcohol consumption in rural western Kenya: A cross-sectional study

**DOI:** 10.1186/s12888-017-1344-9

**Published:** 2017-05-10

**Authors:** Risa Takahashi, Calistus Wilunda, Karani Magutah, Wanja Mwaura-Tenambergen, Boniface Wilunda, Usaneya Perngparn

**Affiliations:** 1 Department of Nursing Science, Faculty of Health Care, Tenri Health Care University, 80-1 Bessho-cho, Tenri City, Nara 632-0018 Japan; 2 College of Public Health Sciences, Chulalongkorn University, Chulalongkorn soi 62, Phyathai Rd, Bangkok, 10330 Thailand; 3 Department of Pharmacoepidemiology, Graduate School of Medicine and Public Health, Kyoto University, Yoshida Konoe-cho, Sakyoku, Kyoto, 606-8501 Japan; 4 Department of Medical Physiology, Moi University, P.O Box 4606-30100, Eldoret, Kenya; 5 Department of Health Systems Management, Kenya Methodist University, P.O. Box 45240-00100, Nairobi, Kenya; 6 United Nations Office on Drugs and Crime, Regional Office for Eastern Africa, P.O. Box 30218-00100, Nairobi, Kenya

**Keywords:** Alcohol use, Hazardous drinking, Substance use, Rural health, Sub-Saharan Africa, Kenya

## Abstract

**Background:**

Studies on alcohol consumption in rural areas in sub-Saharan Africa are scarce. This study aimed to determine the prevalence and determinants of alcohol consumption in rural western Kenya. The study was conducted as a preliminary stage of a community-based intervention to reduce hazardous alcohol consumption.

**Methods:**

A cross-sectional survey of 478 participants aged 18–65 years residing in Ikolomani Sub-county, Kakamega County was conducted in April 2015. Data were collected using an interviewer-administered questionnaire. We defined current drinkers as participants who consumed any alcoholic product in the preceding one month, and hazardous/high-risk drinkers as participants with an Alcohol Use Disorders Identification Test (AUDIT) score of 8 and above. We summarised data using descriptive statistics and used logistic regression to explore for the correlates of each of current alcohol consumption and hazardous/high-risk alcohol consumption.

**Results:**

The sex-standardized prevalence of current alcohol drinkers was 31.7% (95% confidence interval (CI): 26.8%–37.2%). The prevalence was higher in men (54.6%) than in women (8.9%). The mean AUDIT score among current drinkers was 16.9 (SD 8.2) and the sex-standardized prevalence of hazardous/high-risk alcohol drinking was 28.7% (95% CI: 24.1%–34.0%). Traditional brews were the most commonly consumed types of alcohol and most drinkers took alcohol in the homes of alcohol sellers/brewers. In multivariate analyses, the number of drinkers in the family, the number of friends who are drinkers and the attitude towards alcohol intake were positively associated with current alcohol drinking status, and with hazardous/high-risk alcohol consumption. Women were less likely to be current drinkers and hazardous/high-risk drinkers than were men. Other socio-demographic factors were not significantly associated with alcohol consumption.

**Conclusions:**

The prevalence of alcohol consumption in the study area was higher than the national level estimate of 13.3%. The results suggest that the social environment is the main determinant of alcohol consumption in this setting. These findings imply that interventions to mitigate alcohol consumption in this area will have to target the social networks of the alcohol consumers, change the drinkers’ attitude towards alcohol, and tackle the issue of availability of unlicensed homemade brews.

**Electronic supplementary material:**

The online version of this article (doi:10.1186/s12888-017-1344-9) contains supplementary material, which is available to authorized users.

## Background

The third goal of the 17 Sustainable Development Goals (SDGs) focuses on health including mental health and specifies the strengthening, prevention and treatment of the harmful use of alcohol [1]. Harmful alcohol consumption is a major public health problem and a risk factor for poor health globally. Alcohol consumption is the world’s third largest risk factor for disease and disability; in middle-income countries, it is the greatest risk factor for disease, a causal factor in 60 types of diseases and injuries, and a component cause in 200 others [2, 3]. Alcohol abuse is related to the psychological, physical and social health of communities, families and individuals in developed and developing countries [4].

Alcohol-related problems are emerging as major health issues in Africa [5]. Despite this, only a few surveys on alcohol consumption have been conducted in sub-Saharan Africa, especially in rural settings [3, 6, 7] where the economic status, lifestyle, and culture differs from that in urban areas. Most of the available data are disaggregated at the regional level and hence are of little use at the sub-regional/county level: the basic operational level of the health system in Kenya.

In Kenya, reliable data on alcohol consumption and its effects in rural communities are limited, yet consumption of traditional brews is a common practice in many rural parts of the country [5]. Effects of alcohol consumption are emerging in Kenya. A study conducted in Eldoret found that 23.4% of crash-involved patients of motor vehicle accidents were blood alcohol concentration positive and 12.2% were intoxicated [8]. A survey among women in Nairobi found that women who had partners who drink alcohol were significantly more likely to experience both lifetime violence and violence in the preceding year, and those whose partners were intolerable drinkers had a significantly higher reporting of domestic violence compared with those whose husbands drank moderately [9]. Media reports of deaths from consumption of homemade alcohol in Kenya are common [10, 11]. Despite these problems, there is scarce information on the main determinants of alcohol consumption in rural Kenya. Therefore, it is important to understand the prevalence and the main determinants of alcohol drinking to inform the planning of public health intervention strategies to mitigate the problem. This paper presents data on the prevalence and determinants of alcohol consumption in a rural sub-county in western Kenya. The study was conducted as a preliminary stage of an intervention project to reduce hazardous alcohol consumption in the sub-county.

## Methods

### Study design and setting

This cross-sectional study was conducted in Ikolomani Sub-county, Kakamega County, Western Kenya. Ikolomani Sub-county has a population of approximately 104,669 inhabitants [12], a surface area of 143.6 km^2^, and is divided into 4 wards. Subsistence crop and livestock farming are the mainstay economic activities in the area and the predominant ethnic community is the Luhya. Kakamega County is generally composed of a young population: 58% of the population is aged less than 20 years and 37% is aged 20–65 years. A report based on the 2009 Kenya Population and Housing Census data shows that, of the 47 counties in Kenya, Kakamega contributes the highest proportion (4.8%) to the national poverty, and has a poverty incidence of 49.2% [13]. Despite being mainly rural, it is the 7th most densely populated county (546 people/km^2^) in the country [13]. The nearest drug and alcohol rehabilitation centre is located about 100 km away in Eldoret town, Uasin Gishu County [14].

### Sample size and sampling

The study population for the present study is men and women aged 18–65 years residing in Ikolomani Sub-county. According to the National Authority for the Campaign Against Alcohol and Drug Abuse (NACADA), 10.2% of individuals aged 15–65 years in western Kenya currently use alcohol (i.e. use within last 1 month) [15]. Using this information and an alpha value of 0.05 at 95% confidence interval, the minimum required sample size to estimate the prevalence of alcohol consumption was calculated using the Kish Leslie formula [16] to be 478 after further adjustment for a conservative design effect of 3.4.

Multistage sampling using a modified Expanded Program on Immunisation method [17] was used to select study participants. In the first stage, villages were selected by probability proportionate to size. In the second stage, an equal number of individuals to be surveyed in each village was selected. A location near the centre of each selected village was identified and a random walking direction was determined by spinning a pen. Households lying on the transect from the centre to the border of the village were counted and one of them was chosen at random as a starting point. Proximity selection was then used to select subsequent households as the “next nearest” until the desired sample size for the village was attained [18]. In the case of non-response, data collectors replaced the non-responding household with the next one. This method has been validated for use in sampling study subjects in developing countries where it may be difficult to construct a sampling frame [17, 18]. The approach ensures that the sample is self-weighted; eliminating the need to weight the data during analysis. Thirty villages (clusters) were selected. This is the minimum number of clusters that should be selected through this method to ensure sufficient spread of the sample and reduced inter-village variability [18]. Given the sample size of 478, 16 participants were recruited from each village. In each household, all members fulfilling the inclusion criteria were listed. Only one household member per household was interviewed. In case there were two or more eligible members, one of the members was randomly selected.

### Data collection and measurements

Trained data collectors utilising a structured closed-ended questionnaire collected data in April 2015. The questionnaire contained all the questions in the Alcohol Use Disorders Identification Test (AUDIT) tool developed and published by WHO in 1989 [19, 20] (Additional file 1), socio-demographic and economic status questions and concurrent use of tobacco. Other questions included were on current alcohol consumption, type of alcohol consumed, and the presence of other household members who consume alcohol. The questionnaire was translated forth and back from English to Kiswahili. The translated questionnaire was pretested in a neighbouring village. Data collectors had at least secondary level education (i.e. 12 years of education) and were trained prior to data collection.

We defined two outcome variables: current alcohol consumption and hazardous alcohol consumption. A current alcohol consumer referred to one who took any alcoholic product in the past one month [21]. A hazardous/high-risk drinker was defined as a participant with an AUDIT score of 8 and above [22]. The AUDIT was developed based on data collected from a large multinational sample and most studies have found the cut-off score of 8 to have very favourable sensitivity and an acceptable specificity for current ICD-10 alcohol use disorders and future harm [22, 23]. We considered the following variables to be potential determinants of alcohol consumption: age in years (18–29, 30–49, >49), sex, education (none, primary, secondary/higher), wealth index (tertile), number of household members (1–3, 4–6, >6), religion (Roman Catholic, protestant, Muslim), marital status (married/living together, not married), use tobacco (yes, no), sell/prepare alcohol at home (yes, no), number of drinkers in family (0, 1, >1), number of drinking friends (0, 1–5, >5), and alcohol attitude score (tertile). The wealth index was obtained through principal component analysis (PCA) of household assets, access to utilities and type of housing material. Six components were extracted and the first, which explained 25.6% of the variance, was used to represent the wealth index [24]. Likewise, a score on attitude towards alcohol was derived through PCA of 15 Likert-scale questions, adapted from previous unpublished studies, which assessed attitude towards alcohol intake and its effects (Additional file 1). Three components were extracted and the first, which explained 35.5% of the variance, was used to represent an attitude towards alcohol intake. The attitude tool had a high internal consistency (reliability), with a Cronbach’s alpha of 0.851 in a pre-test sample of 30 subjects.

### Statistical analysis

Data were double entered into EpiData (EpiData Association, Odense Denmark) and validated. We used descriptive statistics to summarise the characteristics of participants, the usual place of drinking and types of alcohol consumed. Because our sample contained a disproportionately higher number of women than men, which is contrary to the national population structure, we calculated sex standardised prevalence of current alcohol drinkers and of hazardous/high-risk drinkers. We performed the standardisation using the direct method based on the Kenyan population structure, which consists of about 50% men and 50% women in the age group 15–64 years [25].

To assess the determinants of current alcohol use and hazardous/high-risk alcohol consumption, we performed univariate and multivariate analyses using logistic regression. Variables with *p* < 0.1 in univariate analyses were included in multivariate analysis. We included all subjects in descriptive and univariate analyses but in multivariate analysis, we excluded 19 (4%) subjects who had missing data for either wealth index or attitude score. We report unadjusted and adjusted odds ratios with 95% confidence intervals (CIs) and *p* values. We considered *p* values of less than 0.05 to be statistically significant. All analyses were performed in Stata 12 using survey commands to account for clustering at the village level.

## Results

### Characteristics of participants and the prevalence of current drinking and hazardous/high-risk drinking

The survey included 478 eligible participants, 280 (58.6%) of whom were female, with a mean age of 41 years (Table 1). Only 36.8% of the participants had at least secondary level education, 14% were current tobacco users, 11.9% were from households that sell or prepare alcohol at home, 46.4% had at least one family member who drinks alcohol, and 55.6% had at least one friend who drinks alcohol. The proportion of current tobacco users was higher among men than among women. Men were also more likely to be from households where alcohol was prepared/sold, to have a higher number of drinking friends and family members, and to have a positive attitude towards alcohol than women were. There was little difference in the distribution of men and women by age group, education, wealth index tertile, household size, religion, marital status. The prevalence of both current alcohol consumption and hazardous/high-risk drinking was substantially higher in men than in women (54.6% vs 8.9% and 51.0% vs 6.4%, respectively).

**Table 1:** Characteristics of study participants according to sex

Characteristics	Frequency (%)		
	Male (*n* = 198)	Female (*n* = 280)	Total (*n* = 478)
Age group			
18–29	50 (25.3)	73 (26.1)	123 (25.7)
30–49	70 (35.4)	116 (41.4)	186 (38.9)
50–65	78 (39.4)	91 (32.5)	169 (35.4)
Mean (SD)	42.0 (13.7)	40.6 (14.2)	41 (14.0)
Education			
None	22 (11.1)	43 (15.4)	65 (13.6)
Primary	91 (46.0)	146 (52.1)	237 (49.6)
Secondary/higher	85 (42.9)	91 (32.5)	176 (36.8)
Wealth index tertile (470)			
Rich	62 (32.1)	97 (35.0)	159 (33.8)
Middle	63 (32.6)	92 (33.2)	155 (33.0)
Poor	68 (35.2)	88 (31.8)	156 (33.2)
Household size			
1–3	39 (19.7)	42 (15.0)	81 (17.0)
4–6	93 (47.0)	145 (51.8)	238 (49.8)
> 6	66 (33.3)	93 (33.2)	159 (33.3)
Religion(477)			
Catholic	82 (41.6)	99 (35.4)	181 (38.0)
Protestant	113 (57.4)	181 (64.6)	294 (61.6)
Muslim	2 (1.0)	0 (0.0)	2 (0.4)
Marital status			
Married/living together	142 (71.7)	202 (72.1)	344 (72.0)
Not married	56 (28.3)	78 (27.9)	134 (28.0)
Uses tobacco			
Yes	53 (26.8)	16 (5.7)	69 (14.4)
No	145 (73.2)	264 (94.3)	409 (85.6)
Sell/prepare alcohol at home			
Yes	30 (15.2)	27 (9.6)	57 (11.9)
No	168 (84.9)	253 (90.4)	421 (88.1)
Number of drinkers in family			
0	83 (41.9)	173 (61.8)	256 (53.6)
1	59 (29.8)	73 (26.1)	132 (27.6)
> 1	56 (28.3)	34 (12.1)	90 (18.8)
Number of drinking friends			
0	47 (23.7)	165 (58.9)	212 (44.4)
≤ 5	66 (33.3)	86 (30.7)	152 (31.8)
> 5	85 (42.9)	29 (10.4)	114 (23.9)
Attitude score tertile(467)			
1 (poor attitude)	58 (29.9)	97(35.5)	155 (33.2)
2	52 (26.8)	104(38.1)	156 (33.4)
3 (good attitude)	84 (43.3)	72(26.4)	156 (33.4)

*N* = 478 unless indicated

Table 2 summarises the prevalence of alcohol consumption and hazardous/high-risk drinking by background characteristics according to sex. For both men and women, the prevalence of alcohol consumption and hazardous/high-risk drinking was highest in those with no education, Roman Catholics, current tobacco users, those from households where alcohol is prepared/sold, those with >5 friends who drink, and those with a positive attitude towards alcohol. The sex-standardized prevalence of current alcohol consumption was 31.7% (95% CI: 26.8%–37.2%) while that of hazardous/high risk drinking was 28.7% (95% CI: 24.1%–34.0%). The mean AUDIT score was similar among those who had ever consumed alcohol and the current drinkers: 16.7 (SD 8.1) and 16.9 (SD 8.2), respectively.

**Table 2:** The prevalence of current alcohol consumption and hazardous/harmful alcohol consumption by background characteristics

Characteristics	Current drinker		Hazardous/harmful drinker	
	Male [*n* (%), *n* = 108]	Female [*n* (%), *n* = 25]	Male [*n* (%), *n* = 101]	Female [*n* (%), *n* = 18]
Age group				
18–29	15 (30.0)	3 (4.1)	12 (24.0)	2 (2.7)
30–49	45 (64.3)	12 (10.3)	42 (60.0)	8 (6.9)
50–65	48 (61.5)	10 (11.0)	47 (60.3)	8 (8.8)
Education				
None	17(77.3)	7 (16.3)	17 (77.3)	6 (14.0)
Primary	49(53.9)	14 (9.6)	49 (53.9)	9 (6.2)
Secondary/higher	42(49.4)	4 (4.4)	35 (41.2)	3 (3.3)
Wealth index tertile				
Rich	27 (43.6)	3 (3.1)	21 (33.9)	1 (1.0)
Middle	32 (50.8)	11 (12.0)	32 (50.8)	7 (7.6)
Poor	45 (66.2)	10 (11.4)	44 (64.7)	9 (10.2)
Number of household members				
> 6	37 (56.1)	12 (12.9)	34 (51.5)	8 (8.6)
4–6	55 (59.1)	9 (6.2)	53 (57.0)	7 (4.8)
1–3	16 (41.0)	4 (9.5)	14 (35.9)	3 (7.1)
Religion				
Other	54 (47.0)	6 (3.3)	47 (40.9)	4 (2.2)
Roman Catholic	53 (64.6)	19 (19.2)	53 (64.6)	14 (14.1)
Marital status				
Married/living together	83 (58.5)	13 (6.4)	78 (54.9)	6 (3.0)
Not married	25 (44.7)	12 (15.4)	23 (41.1)	12 (15.4)
Uses tobacco				
No	67 (46.2)	21(8.0)	62 (42.5)	15 (5.7)
Yes	41 (77.4))	4(25.0)	39 (75.0)	3 (20.0)
Sell/prepare alcohol at home				
No	80 (47.6)	17 (6.7)	71 (42.3)	13 (5.1)
Yes	28 (93.3)	8 (39.6)	30 (100)	5 (18.5)
Number of drinkers in family				
0	9 (10.8)	2 (1.2)	9 (10.8)	1 (0.6)
1	51 (86.4)	13 (17.8)	45 (76.3)	8 (11.0)
> 1	48 (85.7)	10 (29.4)	47 (83.9)	9 (26.5)
Number of friends who drink				
0	5 (10.6)	4 (2.4)	5 (10.6)	3 (1.8)
≤ 5	39 (59.1)	13 (15.1)	33 (50.0)	9 (20.7)
> 5	64 (75.3)	8 (27.6)	63 (74.1)	6 (20.7)
Attitude score tertile				
1 (poor attitude)	10 (17.3)	2 (2.1)	9 (15.5)	3 (3.1)
2	28 (53.9)	9 (8.7)	24 (46.2)	6 (5.8)
3 (good attitude)	67 (79.8)	13 (8.8)	65 (77.4)	8 (11.1)

### Usual drinking place and types of alcohol consumed

The most common drinking place for both male (*n* = 108) and female (*n* = 25) drinkers was the alcohol seller’s home [67.6% and 44.0%, respectively (Table 3)]. However, the second most common usual drinking place was own home (32.0%) among women and bar (18.5%) among men. There were no significant differences between men and women in types of alcohol consumed, except for whisky, which was more likely to be consumed by men. The most frequently consumed type of alcohol were homemade brews called *chang’aa* and *busaa*. Most male drinkers (70.4%) consumed *chang’aa* whereas most female drinkers consumed *busaa* (76.0%).

**Table 3:** Usual place of drinking and type of alcohol consumed

	Men [*n* (%), *n* = 108]	Women [*n* (%), *n* = 25]	*p*-value*
Usual place of drinking			0.008
*Changaa/busaa* seller’s home	73 (67.6)	11 (44.0)	
Pub/Bar	20 (18.5)	3 (12.0)	
Respondent’s home	12 (11.1)	8 (32.0)	
Friends home	3 (2.8)	3 (12.0)	
Types of alcohol consumed**			
*Chang’aa*	76 (70.4)	14 (56.0)	0.235
*Busaa*	69 (63.0)	19 (76.0)	0.349
Beer	46 (42.6)	6 (24.0)	0.112
Whiskey	24 (22.2)	1 (4.0)	0.045
Wine	10 (9.3)	0 (0.0)	0.207

*Fisher’s exact test

**The numbers do not add up to the total because multiple responses were allowed

### Determinants of current alcohol consumption

In unadjusted analysis (Table 4), only education, household size, and marital status were not statistically significantly associated with alcohol intake. In adjusted analysis, sex, the number of drinkers in the family, the number of friends who drink, and attitude towards alcohol showed statistically significant associations with alcohol drinking (Table 4). The number of drinkers in the family had the strongest effect on current alcohol consumption followed by attitude score and the number of friends who drink alcohol. Participants with more than one drinker in the family had more than a 35-fold increase in the odds of alcohol intake compared with those who did not have any drinker in the family (OR = 35.11, 95% CI: 10.30–111.75). Those with a positive attitude towards alcohol intake had about eight times higher odds of being alcohol drinkers compared with those with a poor attitude (OR = 7.73, 95% CI: 2.53–23.63).

**Table 4:** Prevalence of current alcohol use and its relationship with socio-demographic variables

Characteristics	Number of drinkers	Unadjusted OR (95% CI)	*P* value	Adjusted OR (95% CI)	*P* value
Age group					
18–29	18	1		1	
30–49	56	2.55 (1.46–4.46)	0.002	2.29 (0.78–6.71)	0.129
> 49	59	3.02 (1.74–5.24)	<0.001	1.89 (0.68–5.23)	0.213
Sex					
Male	108	1		1	
Female	25	0.08 (0.05–0.15)	<0.001	0.07 (0.02–0.18)	<0.001
Education					
None	24	1		--	
Primary	63	0.62 (0.33–1.15)	0.127	--	
Secondary/higher	46	0.59 (0.30–1.18)	0.133	--	
Wealth index tertile					
Rich	30	1		1	
Middle	43	1.71 (0.95–3.08)	0.074	1.45 (0.49–4.29)	0.492
Poor	55	2.42 (1.31–4.46)	0.006	2.12 (0.78–5.72)	0.134
Household size					
> 6	49	1		--	
4–6	64	0.81 (0.50–1.33)	0.400	--	
1–3	20	0.74 (0.40–1.37)	0.324	--	
Religion					
Other*	60			1	
Catholic	72	2.64 (1.63–4.28)	<0.001	2.01 (0.92–4.39)	0.079
Marital status					
Married/living together	96	1		--	
Not married	37	1.00 (0.60–1.64)	0.986	--	
Uses tobacco (478)					
No	88	1		1	
Yes	45	6.92 (3.70–12.93)	<0.001	2.23 (0.63–7.68)	0.209
Sell/prepare alcohol at home					
No	36	1		1	
Yes	97	5.79 (3.28–10.20)	<0.001	1.72 (0.54–5.55)	0.351
Number of drinkers in family					
0	11	1		1	
1	64	20.64 (8.10–52.56)	<0.001	19.91 (6.88–57.65)	<0.001
> 1	58	40.37 (16.38–99.50)	<0.001	35.11 (10.30–119.75)	<0.001
Number of friends who drink					
0	9	1		1	
≤ 5	52	11.50 (5.99–22.09)	<0.001	3.68 (1.61–8.41)	0.003
> 5	72	38.67 (18.98–78.76)	<0.001	5.49 (1.66–18.22)	0.007
Attitude score tertile					
1 (poor attitude)	12	1		1	
2	37	3.71 (1.64–8.37)	0.002	3.66 (1.16–11.51)	0.027
3 (good attitude)	80	12.39 (5.38–28.50)	<0.001	7.73 (2.53–23.63)	0.001

*The entire sample included 113 Protestants and only 2 Muslims

### Determinants of hazardous/high-risk alcohol use

In unadjusted analysis (Table 5), only education, wealth index, and marital status were not significantly associated with hazardous/high-risk alcohol drinking. After adjustment for other factors, sex, the number of drinkers in the family, the number of friends who drink and attitude towards alcohol consumption were associated with hazardous/high-risk alcohol drinking (Table 5). The number of drinkers in the family had the strongest association with hazardous/high-risk alcohol drinking, with the odds of hazardous/high-risk alcohol consumption increasing with the increasing number of drinkers in the family members, the number of friends who drink alcohol, and alcohol altitude score. The determinants of hazardous/high-risk alcohol intake were similar to those of current alcohol consumption.

**Table 5:** Prevalence of hazardous alcohol use and its relationship with socio-demographic variables

Characteristics	Number of drinkers	Unadjusted OR (95% CI)	*P* value	Adjusted OR (95% CI)	*P* value
Age group					
18–29	14	1		1	
30–49	49	2.83 (1.54–5.20)	0.001	2.67 (0.82–8.67)	0.100
> 49	56	3.76 (1.97–7.17)	<0.001	1.97 (0.68–5.81)	0.213
Sex					
Male	101	1		1	
Female	18	0.07 (0.03–0.13)	<0.001	0.08 (0.02–0.18)	<0.001
Education					
None	23	1			
Primary	58	0.60 (0.31–1.13)	0.110	0.33 (0.08–1.37)	0.123
Secondary/higher	38	0.49 (0.23–1.06)	0.068	0.49 (0.10–2.41)	0.371
Wealth index tertile					
Rich	22	1		1	
Middle	39	2.18 (1.15–4.11)	0.018	1.51 (0.53–4.31)	0.431
Poor	53	3.33 (1.64–6.76)	0.001	2.06 (0.73–5.78)	0.165
Household size					
> 6	42	1		--	
4–6	60	0.92 (0.58–1.45)	0.701	--	
1–3	17	0.73 (0.40–1.33)	0.299	--	
Religion					
Other*	51	1		1	
Catholic	67	2.91 (1.76–4.79)	<0.001	1.89 (0.86–4.17)	0.113
Marital status					
Married/living together	84	1		--	
Not married	35	1.10 (0.71–1.70)	0.650	--	
Uses tobacco					
No	76	1		1	
Yes	43	7.32 (4.03–13.31)	<0.001	2.26 (0.63–8.08)	0.205
Sell/prepare alcohol at home					
No	84	1		1	
Yes	35	6.44 (3.73–11.12)	<0.001	1.70 (0.54–5.38)	0.359
Number of drinkers in family					
0	10	1		1	
1	53	16.13 (6.03–43.13)	<0.001	19.62 (6.39–60.23)	<0.001
> 1	56	40.35 (5.65–104.05)	<0.001	37.95 (9.86–146.02)	<0.001
Number of friends who drink					
0	8	1		1	
≤ 5	42	9.59 (4.19–21.98)	<0.001	4.33 (1.63–11.53)	0.004
> 5	69	39.10 (16.90–90.44)	<0.001	6.47 (1.72–24.38)	0.007
Attitude score tertile					
1 (poor attitude)	12	1		1	
2	30	2.82 (1.35–5.87)	0.007	3.18 (1.04–9.75)	0.043
3 (good attitude)	73	10.27 (4.67–22.59)	<0.001	7.03 (2.37–20.82)	0.001

*The entire sample included 113 Protestants and only 2 Muslims

## Discussion

This study provides a snapshot of the prevalence and correlates of alcohol consumption in Kakamega County for the first time. The sex standardised prevalence of current alcohol intake was 31.7%, which is much higher than the official 2012 national level of 13.3% [26] and the prevalence of 9.2% reported in the neighbouring Kisumu County [1]. Factors that could explain these discrepancies include potential underreporting in previous surveys conducted by government agencies (consumption of some traditional brews used to be illegal for a long time [27]) and the true regional variations in the prevalence of alcohol consumption in Kenya. The reported prevalence is, however, similar to that in the neighbouring country of Uganda (28.6%) [7]; the second highest alcohol consumer in Africa [25]. This is not surprising given that Western Kenya borders Eastern Uganda and the residents in the bordering areas have similar ethnic and cultural backgrounds including consumption of traditional alcohol.

The most common types of alcohol consumed in the study area were homemade traditional brews namely, *chang’aa* and *busaa*, which explains why the usual drinking place was the seller’s home. *Chang’aa* is a high alcohol content spirit-like clear drink made by fermenting a mixture of corn/sorghum/millet and sugar for about a week followed by distillation whereas *busaa* is a malt liquor made from fermenting corn flour and sorghum/millet over a shorter period of time (typically two days) [28]. These traditional brews are not standardised, but some studies have estimated their alcohol content to range from 15.3%–34% for *chang’aa* and 3.9%–5.4% for *busaa* [28, 29]. For many years, the Chang’aa Prohibition Act of 1980 [27] prohibited the production, supply, possession, and consumption of *chang’aa,* but not of *busaa,* in Kenya. The Alcoholic Drinks Control Act of 2010 [30] and the Alcoholic Drinks Control Amendment Bill of 2013 [31] repealed the Chang’aa Prohibition Act and focused on production, sale, and licensing rather than on illegalising traditional brews. Thus, traditional brews are legal if the producer is licensed. However, it is difficult for traditional brewers to meet the high standards for licensing as stipulated in the law. The traditional brews are usually not sold in bars or in shops but in people’s homes. One village could contain 3 to 5 such drinking places and this, coupled with affordable prices, ensures that the brews are easily accessible. These results differ from those of another study conducted in a rural setting in eastern Kenya which reported that the most commonly consumed alcohol type was bottled beer (64.8%) followed by local brews [3]. However, this study by Kinoti and colleagues was based on a smaller (*N* = 217) non-random sample, and hence it is subject to a greater degree of random error and selection bias. The price of a half-litre bottle of beer starts from 1.3 US$ (130 Ksh) which is quite expensive for most villagers, but the price of a cup of local brew is only about 0.5 US$; which highlights the issue of affordability of local brews.

The finding of a gender gap in alcohol consumption is similar to what other studies from East Africa have reported [1, 7]. The reasons for the prevalence gap between men and women might be cultural, for instance, gender-based distinctions between male and female based on the traditional system of patriarchy in the community. A qualitative study in the neighbouring Uganda found that alcohol intake among men is associated with masculinity, social dependence and lesser financial empowerment among women [32]. Men have easier access to money than women do. Among rural women, alcohol intake is associated with defiance of the feminine ideals of domesticity [6]. A qualitative study might help to elicit other factors for the gender discrepancy in alcohol intake in the current setting.

Although based on small numbers, we noted some differences in the drinking behaviours between men and women. The proportion of individuals who drank at home was higher among women than among male drinkers. This may be because women in this setting are more likely to be uncomfortable to drink in public places because drinking among women is perceived to be socially undesirable. The most popular drink among women was *busaa* while among men it was *chang’aa*. Women prefer *busaa* over *chang’aa* because of the former’s lower alcohol content [28] and sweeter flavour.

The findings of this study show that having family members and friends who drink is strongly associated with current alcohol drinking status and hazardous/high-risk alcohol drinking. Possible explanation for this can be the influence of peer pressure and easier access to alcohol because friends/family members may buy alcohol for each other. The business of brewing and selling local brews is common in the study setting because of the prevailing poverty situation and the lack of employment opportunities to meet the costs of living including school fees for children. Although these businesses are illegal, they continue to flourish due to limited alternative economic opportunities and probably weaknesses in law enforcement [33].

This study has some limitations. First, some participants, especially women, might have underreported their alcohol consumption due to social desirability and stigma associated with alcohol consumption. Alcohol use by women is a taboo in the study community and women who drink alcohol are held in low esteem. Moreover, as mentioned above, consumption of *chang’aa* was for a long time illegal, which may further affect reporting by both genders. To minimise this bias, data collectors were trained to reassure respondents and to ensure confidentiality before asking questions. Second, the sample size was insufficient to allow precise assessment of multiple correlates of alcohol intake. This may explain why some of the estimates have wider confidence intervals. Third, the measurement of alcohol units in this setting was a challenge because it is difficult to standardise the alcohol content of homemade brews and to estimate the amount consumed. We used the data from a study conducted in 2010 that estimated the alcohol content of traditional brews in western Kenya to be 34% for *chang’aa* and 4% for *busaa* [28]. We also asked participants to estimate their alcohol intake based on measurement containers commonly used by alcohol sellers. Finally, the number of adverse life events, social support, and severity of psychological distress may be associated with hazardous alcohol use, but the present study did not collect data on these variables. Nonetheless, a previous study in Kenya did not find any significant association between these variables and current alcohol intake [6]. Finally, given that this study was conducted in only one sub-county, generalisability of the findings may be limited to other regions with similar socio-cultural and economic profile as the study sub-county.

## Conclusions

In this study conducted in rural western Kenya, we found a prevalence of alcohol intake that is higher than the regional and national levels. Drinkers mainly consumed homemade brews that are cheaper, culturally appropriate and easily accessible. The results on the determinants of alcohol intake suggest that the social environment, rather than an individual’s socio-demographic characteristics, is the main determinant of alcohol consumption in this setting. Although this observation could be due to reverse causation, the findings imply that interventions to mitigate alcohol consumption in this area will have to target the social networks of the alcohol consumers, change the drinkers’ attitude towards alcohol, and tackle the issue of availability of unlicensed traditional brews. Given the resource constraints to tackle the problem of alcoholism in the study area, innovative community-based approaches using locally available resources to mitigate the problem of alcoholism should be piloted and implemented in this and similar contexts. Moreover, the poor economic status in the study area should be addressed and alternative sources of income for traditional alcohol brewers and sellers should be created.

## Additional file

Additional file 1: Attitude towards drinking alcohol and AUDIT tools. (DOCX 105 kb)
