# Supplementary material for: Correlates of alcohol consumption in rural western Kenya: A cross-sectional study
Source: BMC Psychiatry. 2017 May 10;17:175. doi: 10.1186/s12888-017-1344-9 (PMC5424353; doi:10.1186/s12888-017-1344-9)
Supplement: Additional file 1: — Attitude towards drinking alcohol and AUDIT tools. (DOCX 105 kb) [file 12888_2017_1344_MOESM1_ESM.docx]

**Additional file 1**

**Attitude towards drinking alcohol**

**Now I am going to ask you some questions about what you think about alcohol. Please tell me your honest opinion. There is no correct or wrong answer.**

***For each of the following statements, please tell me whether you strongly disagree, disagree, undecided, agree or strongly disagree.***

| Statement | Strongly disagree | Disagree | Undecided | Agree | Strongly agree |
| --- | --- | --- | --- | --- | --- |
| 1. Alcohol enhances social activities? | 1 | 2 | 3 | 4 | 5 |
| 1. Alcohol makes it easier to deal with stress? | 1 | 2 | 3 | 4 | 5 |
| 1. Alcohol makes a connection with peers easier? | 1 | 2 | 3 | 4 | 5 |
| 1. Alcohol gives people something to talk about? | 1 | 2 | 3 | 4 | 5 |
| 1. Alcohol facilitates male bonding? | 1 | 2 | 3 | 4 | 5 |
| 1. Alcohol facilitates female bonding? | 1 | 2 | 3 | 4 | 5 |
| 1. Alcohol enables people to have more fun? | 1 | 2 | 3 | 4 | 5 |
| 1. Alcohol gives people something to do? | 1 | 2 | 3 | 4 | 5 |
| 1. Alcohol makes food taste better? | 1 | 2 | 3 | 4 | 5 |
| 1. Alcohol makes women look sexually attractive? | 1 | 2 | 3 | 4 | 5 |
| 1. Alcohol can make one to become strong? | 1 | 2 | 3 | 4 | 5 |
| 1. Alcohol enhances sexual performance? | 1 | 2 | 3 | 4 | 5 |
| 1. Alcohol makes somebody to think better? | 1 | 2 | 3 | 4 | 5 |
| 1. Alcohol makes somebody to become brave? | 1 | 2 | 3 | 4 | 5 |
| 1. Drinking alcohol prevents malaria infection? | 1 | 2 | 3 | 4 | 5 |

**
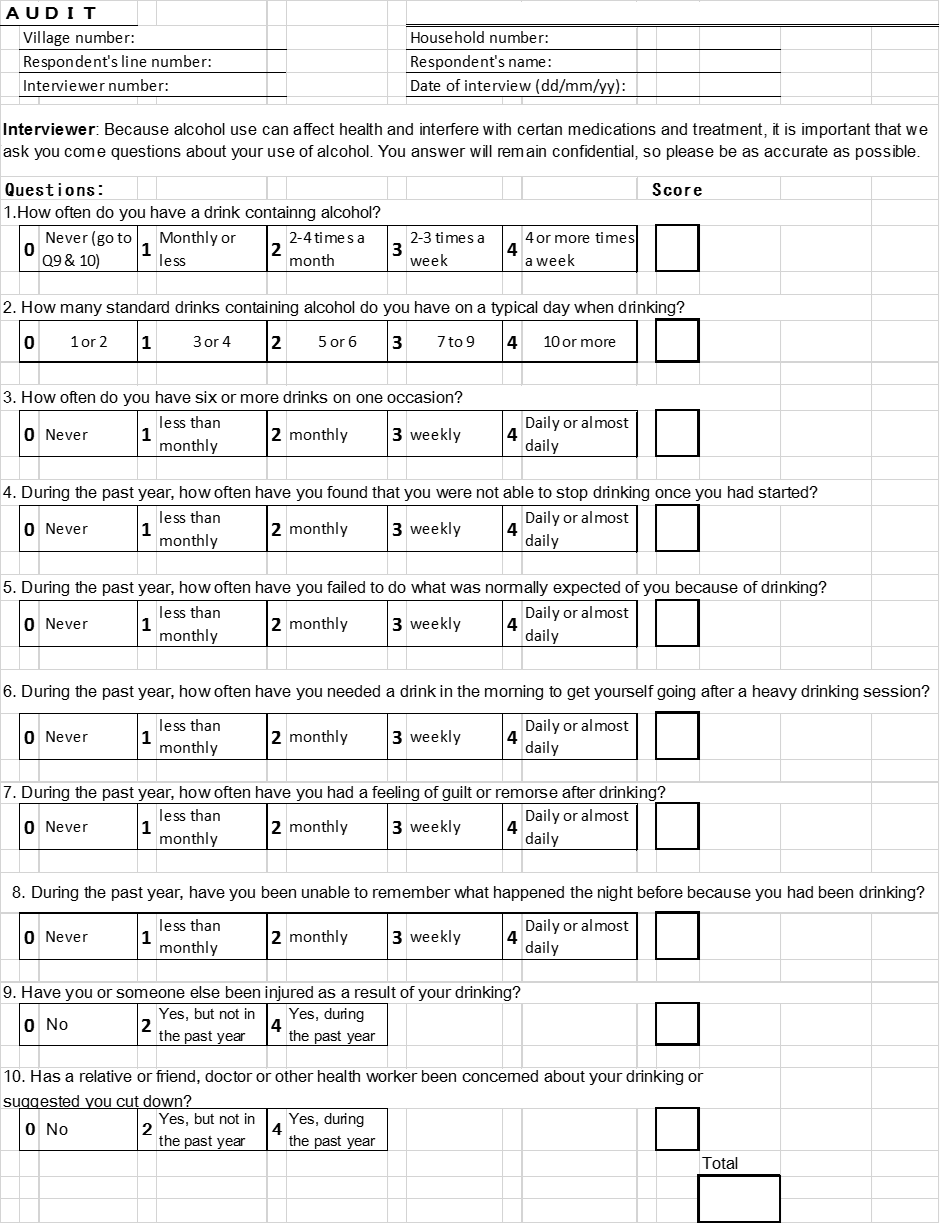
**
